# Biomass and elemental concentrations of 22 rice cultivars grown under alternate wetting and drying conditions at three field sites in Bangladesh

**DOI:** 10.1002/fes3.110

**Published:** 2017-06-15

**Authors:** Gareth J. Norton, Anthony J. Travis, John M. C. Danku, David E. Salt, Mahmud Hossain, Md. Rafiqul Islam, Adam H. Price

**Affiliations:** ^1^ Institute of Biological and Environmental Sciences University of Aberdeen Aberdeen AB24 3UU UK; ^2^ Centre for Plant Integrative Biology School of Biosciences University of Nottingham Sutton Bonington Campus Loughborough LE12 5RD UK; ^3^ Department of Soil Science Bangladesh Agricultural University Mymensingh Bangladesh

**Keywords:** Alternate wetting and drying, arsenic, cadmium, rice, yield, zinc

## Abstract

As the global population grows, demand on food production will also rise. For rice, one limiting factor effecting production could be availability of fresh water, hence adoption of techniques that decrease water usage while maintaining or increasing crop yield are needed. Alternative wetting and drying (AWD) is one of these techniques. AWD is a method by which the level of water within a rice field cycles between being flooded and nonflooded during the growth period of the rice crop. The degree to which AWD affects cultivars differently has not been adequately addressed to date. In this study, 22 rice cultivars, mostly landraces of the *aus* subpopulation, plus some popular improved *indica* cultivars from Bangladesh, were tested for their response to AWD across three different field sites in Bangladesh. Grain and shoot elemental concentrations were determined at harvest. Overall, AWD slightly increased grain mass and harvest index compared to plants grown under continually flooded (CF) conditions. Plants grown under AWD had decreased concentrations of nitrogen in their straw compared to plants grown under CF. The concentration of elements in the grain were also affected when plants were grown under AWD compared to CF: Nickel, copper, cadmium and iron increased, but sodium, potassium, calcium, cobalt, phosphorus, molybdenum and arsenic decreased in the grains of plants grown under AWD. However, there was some variation in these patterns across different sites. Analysis of variance revealed no significant cultivar × treatment interaction, or site × cultivar × treatment interaction, for any of the plant mass traits. Of the elements analyzed, only grain cadmium concentrations were significantly affected by treatment × cultivar interactions. These data suggest that there is no genetic adaptation amongst the cultivars screened for response to AWD, except for grain cadmium concentration and imply that breeding specifically for AWD is not needed.

## Introduction

With an ever‐growing world population, producing sufficient food in the coming decades will be a major focus of crop science. Within in the next 40 years, the world's population is predicted to increase from 7 billion to 9 billion people (Godfray et al. 2010). Rice is expected to play a key role in feeding this increased population. At present, rice provides 20% or more of the daily calorie intake for half of the world's population (Kush 2013). In the future, global rice demand is expected to rise from 676 million tons in 2010 to 852 million tons by 2035 (Kush 2013). Currently, irrigated lowland rice systems represent about 75% of global rice production (Fageria 2007). To produce 1 kg of rice grain, an average of 2500 L of water is needed (Bouman 2009). Globally this equates to one‐third of the World's developed freshwater being used for rice irrigation (Bouman 2009). Not only does rice require large quantities of water, but rice cultivation under flooded conditions also contributes to global methane production (Smith et al. 2008). Therefore, approaches to rice production that require less water, while still maintaining yields are needed. One of the strategies being adopted across parts of Asia is alternate wetting and drying (AWD) (Lampayan et al. 2015). AWD is a technique in which rice fields undergo a number of drying phases during the growing season (Zhang et al. 2009). Farms are encouraged to start with a technique called safe‐AWD, where the water level is allowed to drop 15 cm below the soil surface a number of times during vegetative growth and then the fields are re‐flooded prior to flowering (Lampayan et al. 2015).

If AWD water saving techniques are to be widely adopted, one of the important factors will be the effect that this technique has on grain yield. Some studies have shown AWD has no effect on yield compared to other water management practices (Yao et al. 2012; Howell et al. 2015; Linquist et al. 2015; Shaibu et al. 2015; Liang et al. 2016), some have shown decreases in yield (Sudhir‐Yadav et al. 2012; Linquist et al. 2015; Shaibu et al. 2015) while some show an increase in yield (Yang et al. 2009; Zhang et al. 2009; Wang et al. 2014; Norton et al. 2017). The evidence suggests that “safe‐AWD” has little impact on yield compared to AWD where the soil is allowed to go through more severe periods of drying. In addition, it has been demonstrated that AWD can also reduce the methane emissions from paddy fields (Linquist et al. 2015; Liang et al. 2016).

A key question to address is if there are any cultivar differences in response to AWD. If detected it would suggest breeding efforts will be required specifically targeting AWD rather than traditional flooded conditions. A study by Zhang et al. (2009), using two high yield rice cultivars found that while both AWD treatment and cultivar had significant effects on grain yield there was no interaction between these two factors indicating that both cultivars responded similarly to the AWD treatment. In a study by Howell et al. (2015), two rice cultivars were grown under AWD and continuous flooding (CF) and grain yield was not affected by the cultivar, the AWD treatment or the interaction between cultivar and treatment. These studies suggest cultivars do not differ in response to AWD but a wider survey of rice cultivars is required to be confident this is will hold across the diverse rice germplasm.

The impact of AWD on grain element composition has attracted attention in recent studies because of the potential nutritional value of some elements (e.g., iron and zinc) or the toxic nature of others (arsenic and cadmium) combined with the known effect that soil redox status has on their bioavailability. Several studies have investigated the consequences of AWD on the concentration of single grain elements, for example; grain arsenic decreased under AWD (Somenahally et al. 2011; Linquist et al. 2015; Chou et al. 2016), while zinc (Wang et al. 2014) and cadmium increased under AWD (Yang et al. 2009). The impact that AWD has on a range of grain elements was explored in a single cultivar (Norton et al. 2017). In that study, it was demonstrated that AWD caused an increase in grain manganese (18.5–27.5%), copper (36.7–80.8%), and cadmium (27.8–67.3%) and a decrease in the concentration of sulfur (4.2–15.4%), calcium (6.3–8.7%), iron (10.7–15.5%), and arsenic (13.7–25.7%) compared to plants grown under CF.

It has been demonstrated that there are cultivar differences for the concentration of a large number of elements within the straw and grain of rice (e.g., Jiang et al. 2008; Norton et al. 2010a). The impact that location (field site) has on the accumulation of different elements has previously been investigated for rice (Norton et al. 2010a). In Norton et al. (2010a), 18 different rice cultivars were compared across four different field sites and it was established that all 10 elements measured in the grain showed variation based on cultivar, site and cultivar × site interactions. The identification of variation in grain elements has been exploited for the genetic mapping of genomic regions responsible for grain element concentration (Stangoulis et al. 2007; Lu et al. 2008; Garcia‐Oliveira et al. 2009; Norton et al. 2010b, 2012a,b, 2014; Zhang et al. 2014).

As water saving techniques for rice cultivation become widely adopted, evaluation of the adaptation of cultivars to the different cultivation techniques is needed. In this study, 22 cultivars were tested to determine if the genetic differences between cultivars affected plant mass when grown under AWD compared to CF. In addition, the elemental composition of both straw and grains was determined to identify any effect that AWD has, and to determine if genetics affects the response to AWD treatment. To further explore the effect of different environmental conditions on both the effect of AWD and cultivar differences between cultivars, the same experiment was conducted at three different field sites in Bangladesh.

## Methods

### Rice cultivars

At each site, 22 cultivars were tested (Table 1). The cultivars used in this study are a subset of the cultivars previously genotyped, using a 384 SNP array (Travis et al. 2015). The cultivars were either from the *aus* subpopulation originating from Bangladesh or India, or were improved Bangladeshi cultivars (BR 6, BRRI Dhan 28, and BRRI Dhan 47).

**Table 1:** Cultivars used in this study, including country of origin/collection and subpopulation allocation

Cultivar name	Cultivar identifier	Country of origin/collection	Rice subpopulation^1^
Assam 4 (Boro)	IRGC ID 11482	India	Aus‐1
ARC 5977	IRGC ID 12166	India	Aus‐2
AUS 130	IRGC ID 28984	Bangladesh	Aus‐2
AUS 154	IRGC ID 28997	Bangladesh	Aus‐1
AUS 362	IRGC ID 29149	Bangladesh	Aus‐2
AUS Kushi	IRGC ID 66688	Bangladesh	Aus‐2
Pura Nukna	IRGC ID 26413	Bangladesh	Aus‐admix
Shada Boro	IRGC ID 34752	Bangladesh	Aus‐1
Nai Dumur	IRGC ID 35057	India	Aus‐admix
Dubhi Gora	IRGC ID 74567	India	Aus‐admix
DJ 29	IRGC ID 76316	India	Aus‐1
Jabahul	IRGC ID 86978	Bangladesh	Aus‐admix
Black Gora	GSOR 301017	India	Aus‐admix
Dhala Shaitta	GSOR 301041	Bangladesh	Aus‐2
Kasalath	GSOR 301077	India	Aus‐1
DD 62	GSOR 301306	Bangladesh	Aus‐2
DJ 123	GSOR 301307	Bangladesh	Aus‐admix
DM 59	GSOR 301312	Bangladesh	Aus‐2
ARC 10376	GSOR 301341	India	Aus‐admix
BR 6	–	Bangladesh	Indica
BRRI Dhan 28	–	Bangladesh	Indica
BRRI Dhan 47	–	Bangladesh	Indica

^1^Based on SNP analysis (Travis et al. 2015).

### Field experiment

Three field experiments were conducted during the 2014 boro (dry) season in Bangladesh. The field sites were at Mymensingh (a noncalcareous floodplain soil; 24°42′58′′; 90°25′26′′), Madhupur (a Pleistocene terrace soil; 24°35′19′′; 90°02′22′′) and Rajshahi (a calcareous floodplain soil; 24°23′41′′; 88°31′41′′). Basic soil properties can be found in Table S1. Two different irrigation techniques were tested, and for each treatment four replicate blocks in a randomized block design were used. The water irrigation techniques used were CF and AWD.

For all three field experiments, the rice seeds were sown in a nursery bed at Mymensingh on the 17th December 2013. Prior to transplanting the seedlings at the three field sites, each site was ploughed, and then leveled. The day before transplanting the seedlings into the experimental plots started, the plots were fertilized with 40 kg/ha nitrogen, 15 kg/ha phosphorus, 50 kg/ha potassium, 15 kg/ha sulfur and 3 kg/ha zinc (see Table 2 for dates). A further 40 kg/ha nitrogen (as urea) was supplied during the tiller stage (see Table 2 for dates) and another 40 kg/ha nitrogen at the flowering stage (see Table 2 for dates). The seedlings were transplanted (see Table 2 for dates) into the eight plots at Mymensingh each plot was 22.7 m × 11.8 m, at Rajhashi each plot was 12.4 m × 10 m, and at Madhupur each plot was 24 m × 10 m. Plants were planted in 2 m long rows as two plants per hill with a distance of 20 cm between each hill in a row, there was a 20 cm gap per row. The position of each cultivar in each replicate was randomized. Between each row of test cultivar, a row of a check variety (BRRI Dhan 28) was transplanted.

**Table 2:** Date of sowing, transplanting, start and end of AWD cycles at the three field sites

Date	Rajshahi	Mymensingh	Madhupur
Sowing in seed bed	17.12.2013^1^	17.12.2013^1^	17.12.2013^1^
Transplanted into the field	29.01.2014	05.02.2014–06.02.2014	08.02.2014–09.02.2014
Start of first AWD cycle	20.02.2014	21.02.2014	25.02.2014
Start of second AWD cycle	05.03.2014	05.03.2014	07.03.2014
Start of third AWD cycle	19.03.2014	15.03.2014	16.03.2014
Start of fourth AWD cycle	31.03.2014	27.03.2014	27.03.2014
End of AWD cycles	13.04.2014	15.04.2014	12.04.2014
Harvest	07.05.2014–18.05.2014	08.05.2014–28.05.2014	07.05.2014–31.05.2014
Initial fertilizer application	28.01.2014	04.02.2014	07.02.2014
First split nitrogen split	21.02.2014	27.02.2014	01.03.2014
Second split nitrogen split	22.03.2014	27.03.2014	30.03.2014

AWD, Alternative wetting and drying.

^1^All seeds were sown in seed beds in Mymensingh and transported to the other two sites.

After the plants were transplanted, the plots were flooded. For the four CF plots, the surface water was kept at a depth of between 2 and 5 cm above the soil surface during the vegetative stage and reproductive stage. For the four AWD plots, plastic perforated tubes (pani pipe) were placed across the plots to monitor the depth at which the soil was saturated with water. The objective was to allow water to drain/percolate naturally from the AWD plots until the average depth of the water was 15 cm below the soil surface, at which point the plots were irrigated to bring the water depth to between 2 and 5 cm above the soil surface. At each site, the AWD plots went through four cycles of soil drying (Table 2). After the final cycle, the AWD plots were kept flooded and maintained the same as the CF plots.

After the cultivars had flowered and the grain matured, the grains and straw from each cultivar was harvested by hand from the six central hills of each row. The grain was then threshed by hand and the grain weighed to determine grain mass. Grain mass is expressed as the mass of grains harvested from the six central hills. The straw was harvested approximately 5 cm above the soil, dried and then weighed to determine the straw weight. Straw mass is expressed as the mass of straw harvested from the six central hills. When dry, the straw was cut into small pieces ~1–2 cm long. A subsample of grains and straw was then sent to the University of Aberdeen, UK for elemental analysis.

### Rice straw and grain analysis

Elemental analysis of rice straw and grains were conducted as described in Norton et al. (2017). Briefly rice grains were dehusked and oven dried, followed by microwave digestion with concentrated HNO_3_ and H_2_O_2_ as described in Norton et al. (2012a). Straw was oven dried, powdered, and digested using nitric acid and hydrogen peroxide on a block digester (Norton et al. 2017). Total elemental analysis (sodium, magnesium, phosphorus, potassium, calcium, chromium, manganese, iron, cobalt, nickel, copper, zinc, arsenic, molybdenum, and cadmium) was performed by inductively coupled plasma – mass spectroscopy. Trace element grade reagents were used for all digests, and for quality control replicates of certified reference material (Oriental basma tobacco leaves [INCT‐OBTL‐5]) and rice flour [NIST 1568b]) were used; blanks were also included. All samples and standards contained 10 *μ*g/L indium as the internal standard. In addition to elemental analysis on digested material described above, the concentration of nitrogen and carbon were determined on the powdered samples using an NCS analyser (NA2500 Elemental Analyser; Carlo Erba Instruments Wigan, UK).

### Statistical analysis

All statistical analyses were performed, using the statistical software Minitab v.17 (State College, PA) and SigmaPlot v.13 (Systat Software Inc., San Jose, CA, USA). For the plant mass traits and the plant elemental concentration traits, a three‐way ANOVA was conducted with treatment (AWD and CF), site and, cultivar as the explanatory variables. For the three‐way ANOVA, the presence of an interaction between the three explanatory variables was also determined. For correlation analysis, a Spearman’s rank correlation was used.

## Results

**Figure 1:**
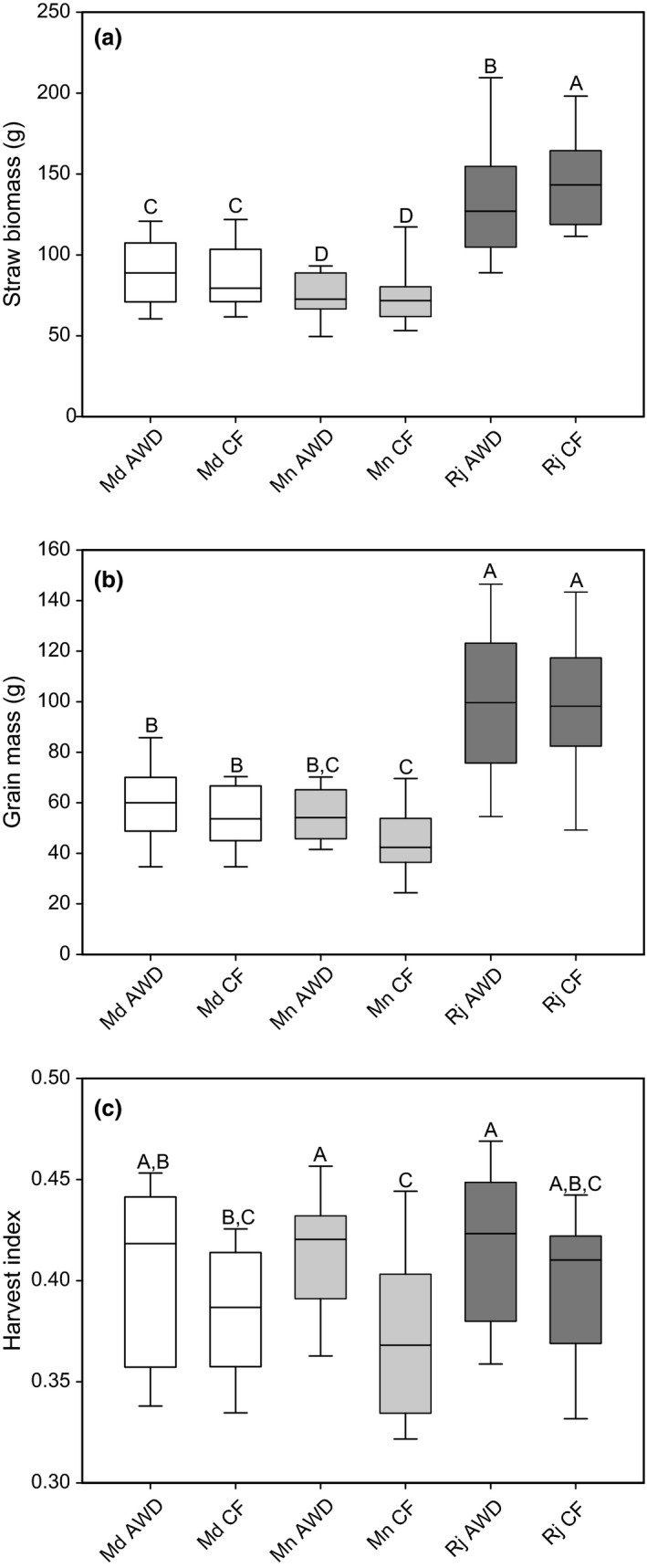
Yield traits for the 22 cultivars grown at the three different sites under AWD and CF. (a) straw biomass, (b) grain mass, (c) harvest index. Md = Madhupur, Mn = Mymensingh, Rj = Rajshahi. Data which had the same letter are not significantly different. Grain and straw mass are the sum of the mass from the six hills. AWD, Alternative wetting and drying; CF, continuous flooding.

The mean data for each of the cultivars grown under the different water treatments at each site are presented in Table S2. Graphs presenting the effect of treatment and site for all traits measured are provided in Figures 1, 3, 4, 5 and Figures S1, S2.

### Straw biomass

**Figure 2:**
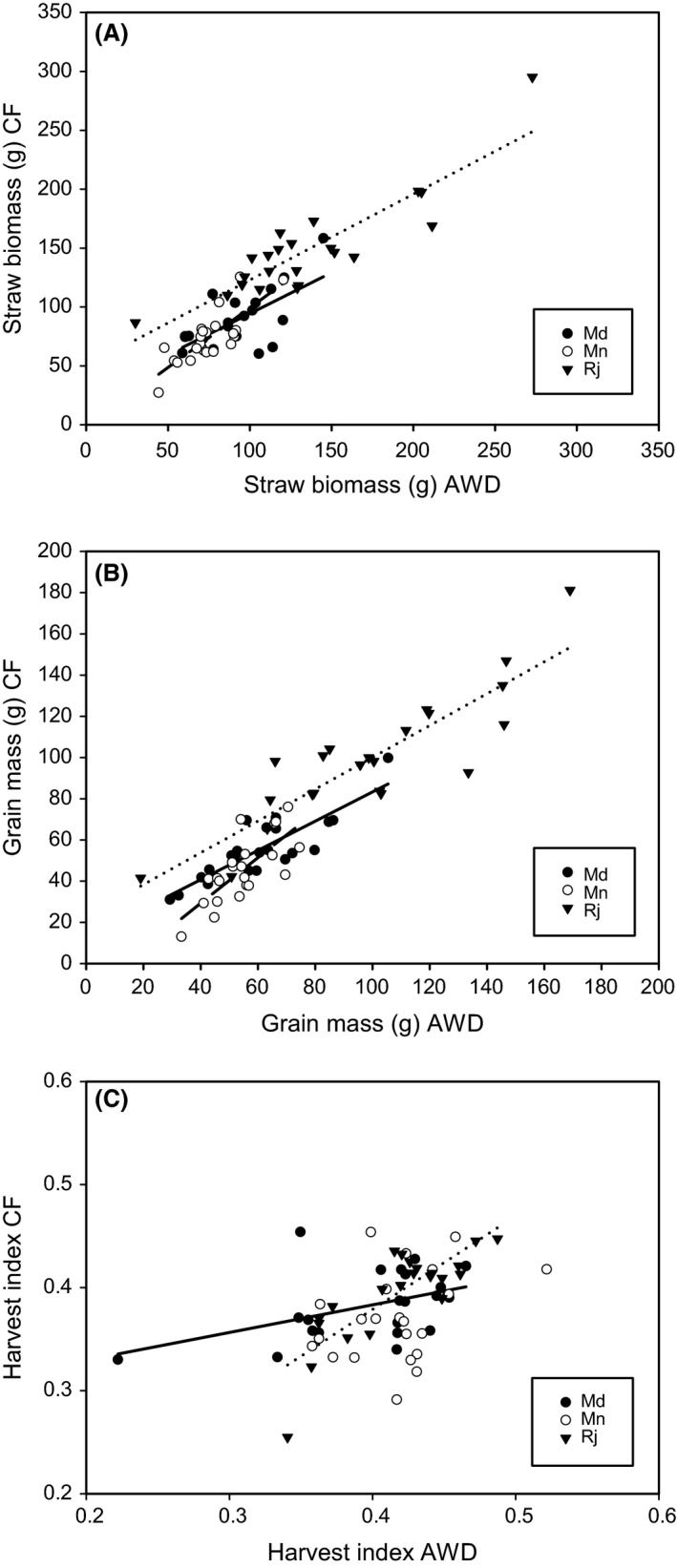
Correlation between yield components of the cultivars grown under AWD and CF conditions at the three field sites. (A) straw biomass, (B) grain mass, (C) harvest index. Filled circles (and solid line) are for the cultivars grown at Md, open circles (and dashed line) are for cultivars grown at Mn, and upside down filled triangles (and dotted line) are for cultivars grown at Rj. Only significant correlations (*P* < 0.05) are indicated with lines. For straw biomass at each site, there was a significant relationship between AWD and CF, Mn *r* ^2^ = 55.2%, Md *r* ^2^ = 71.7%, Rj *r* ^2^ = 77.2%. For grain mass at each site, there was a significant relationship between AWD and CF (*P* < 0.001), Mn *r* ^2^ = 70.3%, Md *r* ^2^ = 52.9%, Rj *r* ^2^ = 76.2%. For harvest index, there were a significant relationship between AWD and CF at two of the three sites Md *r* ^2^ = 20.8%, Rj *r* ^2^ = 67.4%. Grain and straw mass are the sum of the mass from the six hills. AWD, Alternative wetting and drying; CF, continuous flooding.

There was a significant difference between the three field sites for straw biomass and a site by treatment interaction (*P* < 0.001; Table 3). The highest straw biomass was at Rajshahi (mean 142.3 g), followed by Madhupur (89.0 g) and the lowest average at Mymensingh (74.3 g) (Fig. 1a). Overall, there was no significant difference in straw biomass for plants grown under AWD compared to CF. However, there was a significant difference at the Rajshahi site, where plants grown under CF had a higher straw biomass compared to those grown under AWD. There were significant differences in straw biomass between cultivars and significant two‐way interactions for site by treatment interaction and cultivar by site interaction (Table 3). There was a significant positive correlation between the straw biomass of cultivars grown under AWD when compared to CF for each of the three field sites (Fig. 2A).

**Table 3:** Statistical analysis of yield traits across the three different site for the 22 cultivars grown under AWD and CF (treatment). Values reported are the *f*‐values from the ANOVA with the asterisk indicating the level of significance

Trait	Site (S)df = 2	Treatment (T)df = 1	Cultivar (C)df = 21	S × Tdf = 2	S × Cdf = 42	T × Cdf = 21	S × T × Cdf = 42
Straw biomass (g)	315.66***	NS	22.61***	4.78**	4.95***	NS	NS
Grain mass (g)	263.85***	6.23*	17.62***	NS	3.81***	NS	NS
Harvest index	3.56*	35.26***	7.20***	3.15*	2.09***	NS	NS

AWD, Alternative wetting and drying; CF, continuous flooding; NS, not significant.

*P < 0.05, **P < 0.01, ***P < 0.001.

### Grain mass

There was a significant difference (*P* < 0.001) for grain mass between the three field sites, with the highest grain mass being at Rajshahi (mean 99.6 g), followed by Madhupur (58.3 g) and lowest at the Mymensingh (50.6 g) (Fig. 1b). Overall, there was a significant difference (*P* = 0.02) in grain mass for plants grown under AWD compared to CF, with the AWD plants on average having a grain mass of 71.7 g compared to 67.3 g for the CF plants. There was no significant site by treatment interaction. There was, however, a significant difference (*P* < 0.001) in grain mass between the cultivars and a significant (*P* < 0.001) cultivar × site interaction (Table 3). There was a significant positive correlation between the grain mass of cultivars grown under AWD when compared to CF for each of the three field sites (Fig. 2B).

The data reported is the grain mass per row of six cultivars. This can be converted into an approximation of grain yield, by scaling up the value based on the planting density, however this must be used with caution. The yields at the three sites under the two different treatments are as follows: 3.8 t/ha for Mymensingh CF, 4.5 t/ha for Mymensingh AWD, 4.6 t/ha for Madhupur CF, 5.0 t/ha for Madhupur AWD, 8.3 t/ha for Rajshahi CF, and 8.3 t/ha for Rajshahi AWD.

### Harvest index

There was a significant difference in harvest index for the three sites, with plants grown at the site in Rajshahi having the highest harvest index and plants grown at the Madhupur site having the lowest harvest index (Fig. 1c). Overall the plants grown under AWD had a greater harvest index than those grown under CF conditions. However, this was only a small effect, with harvest index increasing on average by 6.9% under AWD. There was a site × treatment interaction revealing that the positive effect of AWD on harvest index was much stronger in Mymeningh than the other sites (Fig. 1c). There was also a significant cultivar difference for harvest index and site × cultivar interaction (Table 3). There were significant positive correlations between the harvest indexes of the cultivars grown under AWD when compared to CF at Madhurpur and Rajhashi, but not at Mymensingh (Fig. 2C).

### Straw carbon and nitrogen concentration

The concentration of carbon in the rice straw was not affected by treatment, but it was significantly affected by the site where the rice plants were grown (Table 4), although the effect was small. The straw biomass of rice plants grown at Madhupur and Mymensingh were approximately 43% carbon while the plants grown at Rajshahi were approximately 41.5% carbon on average. There was also a significant cultivar difference in the concentration of carbon measured in the cultivars across all sites and treatments (Table 4).

**Table 4:** Statistical analysis of straw element traits across the three different site for the 22 cultivars grown under AWD and CF (treatment). Values reported are the *f*‐values from the ANOVA with the asterisk indicating the level of significance

Trait	Site (S)df = 2	Treatment (T)df = 1	Cultivar (C)df = 21	S × Tdf = 2	S × Cdf = 42	T × Cdf = 21	S × T × Cdf = 42
N	48.64***	46.34***	12.39***	10.00***	1.65**	NS	NS
C	28.96***	NS	1.70*	NS	NS	NS	NS
Na	18.32***	NS	7.03***	5.26**	NS	NS	NS
Mg	66.30***	6.77*	7.64***	NS	NS	NS	NS
P	25.46***	22.05***	4.22**	4.70*	1.44*	NS	NS
K	118.02***	NS	4.94***	3.18*	NS	NS	NS
Ca	6.04**	NS	3.56***	NS	1.47*	NS	NS
Cr	8.84***	13.12***	NS	4.22*	1.74**	NS	NS
Mn	154.10***	NS	2.80***	4.49*	NS	NS	NS
Fe	NS	17.59***	2.64***	7.03**	1.60*	NS	NS
Co	54.87***	7.47**	5.88***	NS	NS	NS	NS
Ni	NS	7.52**	NS	3.58*	1.72**	NS	NS
Cu	24.45***	7.71**	8.95***	NS	1.44*	NS	NS
Zn	123.80***	NS	NS	NS	NS	NS	NS
As	75.01***	27.56***	2.79***	23.74***	1.82**	NS	NS
Mo	NS	16.52***	NS	7.98***	NS	NS	NS
Cd	50.94***	NS	NS	9.96***	NS	NS	NS

AWD, Alternative wetting and drying; CF, continuous flooding; NS, not significant.

*P < 0.05, **P < 0.01, ***P < 0.001.

**Figure 3:**
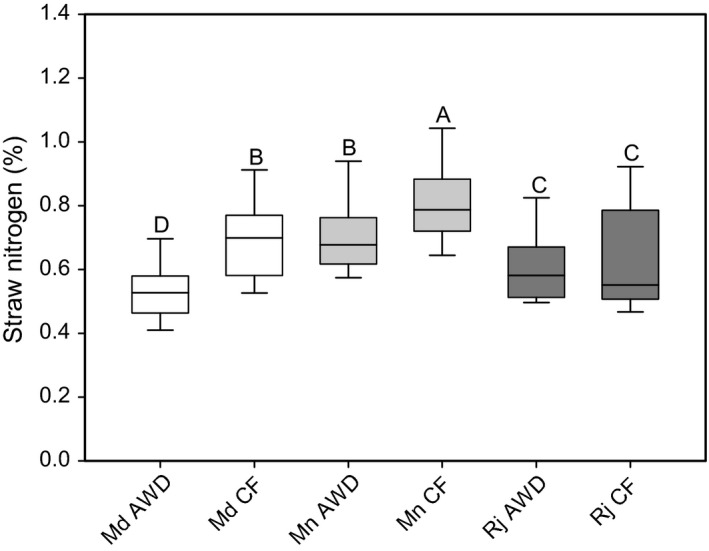
Straw nitrogen for the 22 cultivars grown at the three different sites under AWD and CF. Md = Madhupur, Mn = Mymensingh, Rj = Rajshahi. Data which has the same letter are not significantly different. AWD, Alternative wetting and drying; CF, continuous flooding.

The concentration of straw nitrogen was significantly affected by the site where the rice plants were grown, if they were grown under AWD or CF and there was a significant difference between the cultivars (Table 4; Fig. 3). A higher average nitrogen concentration in the straw was observed at the Mymensingh site (0.8%), while the average nitrogen concentration in the straws of rice plants was the same at both the Madhupur and Rajshahi sites (0.6%). On average, AWD decreased straw nitrogen (~0.6%) compared to CF (~0.7%) across all sites and cultivars. There were also significant effects on straw nitrogen concentration for site × treatment and site × cultivar interactions (Table 4). AWD caused a significant decrease in straw nitrogen compared to the CF treatment when the Madhupur and Mymensingh sites were examined individually (Fig. 3).

### Straw macro and micro elemental concentration

#### Straw arsenic

**Figure 4:**
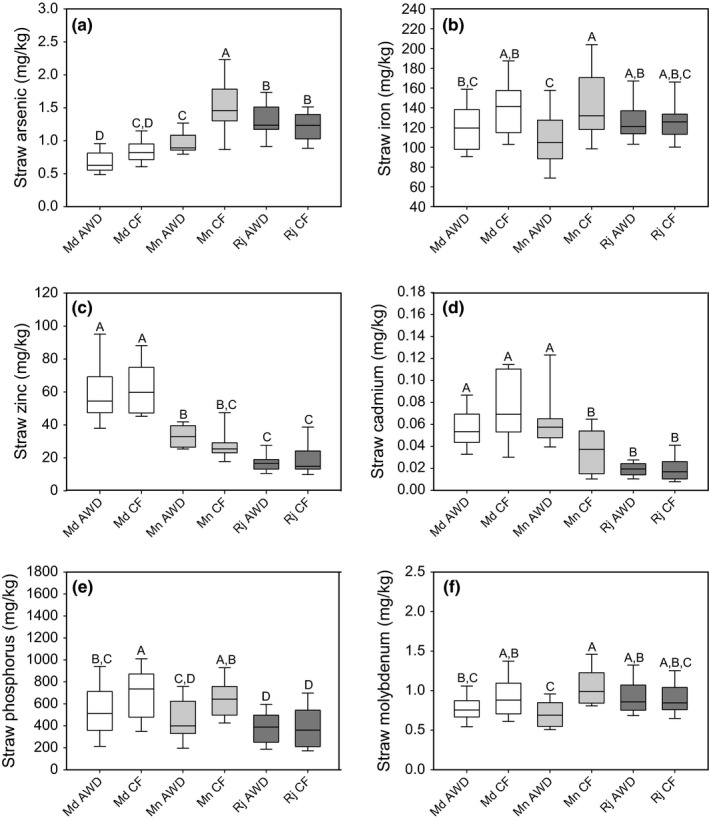
Straw element traits for the 22 cultivars grown at the three different sites under AWD and CF. (a) grain arsenic, (b) grain iron, (c) grain zinc, (d) grain cadmium, (e) grain phosphorus, (f) grain molybdenum. Md = Madhupur, Mn = Mymensingh, Rj =  Rajshahi. Data which has the same letter are not significantly different. AWD, Alternative wetting and drying; CF, continuous flooding.

The concentration of straw arsenic was significantly affected by the site where the plants were grown (Table 4). The average straw arsenic concentration was higher at Rajshahi and Mymensingh (1.27 and 1.25 mg/kg, respectively) compared to Madhupur (0.76 mg/kg). Overall, AWD significantly decreased straw arsenic by 16.7% compared to CF. However, when the AWD treatment effect was explored at each site individually, there was only a significant difference between the AWD and CF treatments at the Mymensingh site, with a 35% decrease in grain arsenic in the AWD treatment (Fig. 4a). There was also a significant cultivar difference in the concentration of straw arsenic measured in cultivars across all sites and treatments (Table 4).

#### Straw iron

The concentration of straw iron was not significantly affected by the site where the plants were grown (Table 4). There was an overall treatment effect across all sites, with AWD on average causing a 12.9% decrease in straw iron compared to CF. However, when each site was examined separately, there was only a significant difference in straw iron at the Mymensingh site, with plants grown under AWD having on average 25% less iron in the straw (Fig. 4b). There was a significant cultivar difference in the concentration of straw iron measured in the cultivars across all sites and treatments (Table 4), as well as a site × cultivar interaction for straw iron.

#### Straw zinc

The only significant factor that affected zinc concentration in the straw was the site at which the plants were grown (Table 4). The highest concentrations of zinc in the straw were found in plants grown at the Madhupur site (61.04 mg/kg) followed by Mymensingh (30.19 mg/kg), with the lowest average concentration at the Rajshahi site (18.54 mg/kg) (Fig. 4c).

#### Straw cadmium

The concentration of straw cadmium was significantly affected by the site where the plants were grown (Table 4), with the average straw cadmium concentration being the highest at Madhupur (0.07 mg/kg) followed by Mymensingh (0.05 mg/kg) and Rajshahi having, on average, the lowest (0.02 mg/kg). Overall, across all sites, the AWD treatment did not affect cadmium concentration in the straw. However, there was a significant site × treatment interaction and when each site was examined separately there was a significant difference in straw cadmium at the Mymensingh site, with the plants grown under AWD having on average 70% more cadmium in straw (Fig. 4d). There was no significant cultivar difference in the concentration of straw cadmium measured in cultivars across all sites and treatments (Table 4).

#### Straw phosphorus

The concentration of straw phosphorus was significantly affected by site, treatment, and cultivar as well as interactions between site × treatment and site × cultivar (Table 4). The site with the highest phosphorus concentration across treatments was Madhupur (631.8 mg/kg), followed by Mymensingh (550.3 mg/kg), and Rajshahi having on average the lowest straw phosphorus concentration (390.5 mg/kg). The AWD treatment caused a significant decrease in straw phosphorus across all sites (AWD average concentration 459 mg/kg and CF average concentration 590 mg/kg). There was no significant effect of AWD treatment at the field site in Rajshahi, but AWD caused a significant decrease in straw phosphorus compared to CF at the Madhupur (26.3%) and Mymensingh (29.9%) sites (Fig. 4e).

#### Straw molybdenum

Two significant factors affected the concentration of molybdenum in the straw. There was an overall AWD treatment effect across sites and a significant interaction between site × treatment (Table 4). AWD caused a 17.5% decrease in straw molybdenum compared to the plants grown under CF across all sites. AWD treatment only caused a significant effect at the Mymensingh site, with an average straw molybdenum concentration for the plants grown under AWD being 0.71 mg/kg compared to 1.09 mg/kg in plants grown under CF (Fig. 4f).

#### Other elements in the straw

The site where the rice was grown had a significant effect on elemental concentration in the straw for all other elements measured (Table 4). Across all sites and cultivars, the AWD treatment caused a significant change in the concentration of magnesium, chromium, cobalt, nickel, and copper (Table 4, Figure S1). Across the sites and treatments, cultivar caused a significant differences in straw concentration of sodium, magnesium, potassium, calcium, manganese, cobalt, and copper (Table 4). A number of two way interactions between site × treatment and site × cultivar were identified for some elements, however, no treatment × cultivar interactions or three‐way site × treatment × cultivar interactions were detected.

### Macro and micro elemental concentrations in the grains

#### Grain arsenic

**Figure 5:**
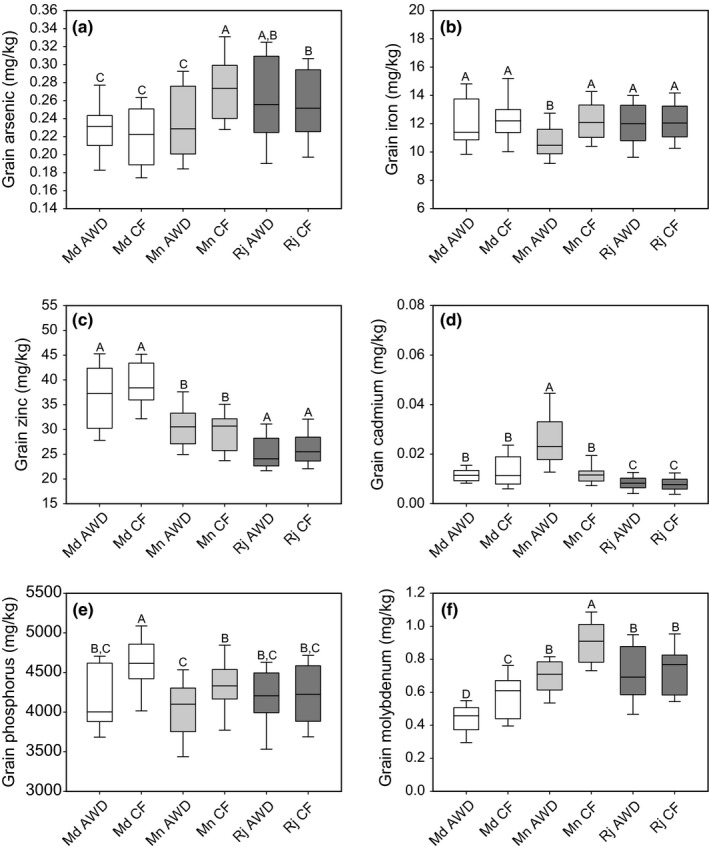
Grain element traits for the 22 cultivars grown at the three different sites under AWD and CF. (a) grain arsenic, (b) grain iron, (c) grain zinc, (d) grain cadmium, (e) grain phosphorus, (f) grain molybdenum. Md = Madhupur, Mn = Mymensingh, Rj = Rajshahi. Data which has the same letter are not significantly different. AWD, Alternative wetting and drying; CF, continuous flooding.

There were significant effects of site, treatment, cultivar, and a site × treatment effect for grain arsenic (Table 5) with AWD on average decreasing grain arsenic by 3.2% across all sites. There was only a significant difference between AWD and CF at the Mymensingh site, with a 16.0% decrease in grain arsenic in the AWD treatment (Fig. 5a).

**Table 5:** Statistical analysis of grain element traits across the three different site for the 22 cultivars grown under AWD and CF (treatment). Values reported are the *f*‐values from the ANOVA with the asterisk indicating the level of significance

Trait (mg/kg)	Site (S)df = 2	Treatment (T)df = 1	Cultivar (C)df = 21	S × Tdf = 2	S × Cdf = 42	T × Cdf = 21	S × T × Cdf = 42
Na	35.89***	17.7***	14.78***	12.84***	1.47*	NS	NS
Mg	NS	33.83***	6.03***	3.67*	NS	NS	NS
P	6.73**	27.64***	6.10***	5.67**	NS	NS	NS
K	23.95***	5.05*	3.27***	8.85***	NS	NS	NS
Ca	13.66***	6.78*	16.32***	3.34*	NS	NS	NS
Cr	NS	NS	NS	NS	NS	NS	NS
Mn	749.00***	NS	9.56***	30.68***	1.84**	NS	NS
Fe	10.12***	22.99***	12.27***	7.77**	1.95**	NS	NS
Co	146.96***	3.95*	23.21***	25.54***	1.57*	NS	NS
Ni	132.45***	5.68*	2.37*	NS	NS	NS	NS
Cu	282.03***	63.78***	11.73***	48.36***	1.64*	NS	NS
Zn	301.60***	NS	19.39***	4.28*	1.69*	NS	NS
As	30.46***	4.22*	12.70***	16.53***	NS	NS	NS
Mo	110.09***	56.53***	6.24***	11.02***	1.77*	NS	NS
Cd	150.71***	74.95***	13.19***	91.81***	2.31***	2.59***	5.12***

AWD, Alternative wetting and drying; CF, continuous flooding; NS, not significant.

*P < 0.05, **P < 0.01, ***P < 0.001.

#### Grain iron

The site where the plants were grown, the AWD treatment they received and the cultivar all had a significant effect on grain iron concentration (Table 5). In addition, significant effects on grain iron concentration were observed for both site × treatment and site × cultivar interactions. Overall, the plants with the lowest grain iron concentration were grown at Mymensingh (11.4 mg/kg) while the other two sites had a higher concentration of grain iron (12.1 and 12.2 mg/kg, for Rajshahi and Madhupur, respectively). Overall, AWD decreased grain iron by 5.8% across site and cultivars. There was only a significant difference between AWD and CF treatments at the Mymensingh site, where AWD caused a 12.4% decrease in grain iron (Fig. 5b).

#### Grain zinc

The site where plants were grown and the cultivar had a significant effect on grain zinc concentration, but there was no overall AWD treatment effect (Table 5). The site with the lowest average grain zinc concentration across both AWD and CF treatments was Rajshahi (25.8 mg/kg), followed by Mymensingh (30.2 mg/kg) and the site with the highest grain zinc concentrations was Madhupur (average 37.8 mg/kg). There was no significant effect of AWD treatment on grain zinc compared to CF at any of the three sites across all of the cultivars (Fig. 5c).

#### Grain cadmium

The site where plants were grown, the AWD treatment they received and the cultivar all had a significant effect on grain cadmium concentration (Table 5). In addition, significant two‐way interactions affecting grain cadmium concentration were observed for all combinations of factors (site, treatment and genotype) as well as a significant three‐way interaction between all of the factors. AWD caused a 43% increase in grain cadmium compared to the CF treatment on average across the three sites. A significant difference in grain cadmium between the AWD and CF‐treated plants was only observed at the Mymensingh site (Fig. 5d).

#### Grain phosphorus

The site where plants were grown, the AWD treatment they received and the cultivar all had significant effects on grain phosphorus concentration (Table 5). The plants grown at Madhupur had significantly higher concentrations of grain phosphorus (4390 mg/kg) compared to Mymensingh (4197 mg/kg) and Rajshahi (4191 mg/kg). AWD reduced grain phosphorus by 5.9% compared to plants grown under CF across all three sites. There was a significant decrease in grain phosphorus concentration between AWD treatments at the Madhupur (9.8%) and Mymensingh (6.6%) site (Fig. 5e).

#### Grain molybdenum

The site where plants were grown, the AWD treatment they received and the cultivar all had significant effects on grain molybdenum concentration (Table 5). In addition, significant effects on grain molybdenum concentration were observed for site × treatment and site × cultivar interactions. AWD significantly decreased grain molybdenum concentration by 16.9% across all sites. Plants with the lowest grain molybdenum were grown at the Madhupur site (0.50 mg/kg). While those with the highest grain molybdenum concentration were grown at the Mymensingh site (0.79 mg/kg). However, AWD also significantly decreased grain molybdenum at Mymensingh (22.8%) and at Madhupur (25.6%) (Fig. 5f).

#### Other elements in the grain

For all the other elements measured except magnesium and chromium, the site where the rice was grown had a significant effect on elemental concentration in the grains (Table 5, Fig. 2). Across all the sites and cultivars, the treatment AWD caused a significant change in the concentration of sodium, magnesium, potassium, calcium, cobalt, nickel, and copper. There were significant cultivar effects between cultivars for the concentration of all elements measured in the grain except chromium across all sites and treatments. A number of two‐way site × treatment and site × genotype interactions were also detected (Table 5).

### Impact of flowering time on grain traits

At the Mymensingh field site, the flowering time of the accessions was monitored. This was not done at the other two sites. At this site, all the 22 accessions initiated flowering prior to the last AWD cycle finishing. There was variation in flowering time with the earliest flowering cultivars initiating flowering 17 days before the AWD cycles finished and latest flowering cultivar initiating flowering 3 days prior to the last AWD cycle finishing. Under both AWD and CF flowering time did not significantly correlate with grain mass, straw biomass, or harvest index. For the plant grown under AWD conditions, a number of grain elements concentration correlated with flowering time; there was a positive correlation for grain nickel (*r* = 0.600, *P* = 0.004) and negatively correlations with grain phosphorous (*r* = −0.452, *P* = 0.040), grain zinc (*r* = −0.499, *P* = 0.021) and grain arsenic (*r* = −0.629, *P* = 0.002) (Figure S3). For the plants grown under CF, flowering time also correlated with a number of grain elements; there were negative correlations for grain magnesium (*r* = −0.515, *P* = 0.017), grain phosphorus (*r* = −0.577, *P* = 0.006), and grain zinc (*r* = −0.450, *P* = 0.041). To compare if the impact of flowering time differed across treatments, for each cultivar, the ratio of the AWD concentration for each element was compared to the concentration of the element in the cultivar grown under CF. This ratio was then tested for its relationship with flowering time (Figure S3). Only the ratio for arsenic significantly correlated with flowering time (*r* = −0.510, *P* = 0.018).

## Discussion

One of the most important aspects of the adoption of a rice cultivation practice will be the impact that cultivation practice has on yield. Previous studies on the effect of AWD on yield compared to other rice cultivation practices have reported varying impacts from a reduction in yield to increases in yield (Yang et al. 2009; Zhang et al. 2009; Sudhir‐Yadav et al. 2012; Yao et al. 2012; Wang et al. 2014; Howell et al. 2015; Linquist et al. 2015; Shaibu et al. 2015; Norton et al. 2017). In this study, plants grown under AWD across all three sites did show an increase in grain mass. However, at each individual field site, different magnitudes of treatment effects were observed. The AWD treatment effects were greatest in Mymensingh but no significant effects were observed in Rajshahi. Straw biomass was not consistently affected by AWD. The largest and most consistent effect on yield traits was for the harvest index. A significant difference was observed at the Mymensingh site with plants grown under AWD having an increased harvest index, and at the other two sites a similar trend was also observed. This may indicate that there is a change in the allocation of resources for plants grown under AWD. It has been shown in a number of studies that either the number of tillers or productive tillers increases in plants grown under AWD (Yang and Zhang 2010; Howell et al. 2015; Norton et al. 2017). As part of this field experiment, the number of productive tillers was determined for a single cultivar (these results are presented in Norton et al. (2017)). It was observed that plants grown under AWD had a greater number of productive tillers. A similar process may contribute to effects on harvest index in other cultivars observed in this study.

A few previous studies have tested a small number of rice cultivars to identify variation in yield trait responses to AWD (e.g., Zhang et al. 2009; Howell et al. 2015). In this study, 22 cultivars were tested for their yield response under AWD compared to CF. Significant cultivar differences were identified for yield as well as a significant interaction between cultivar and the site at which plants were grown, but importantly no significant interactions between cultivar and treatment were identified for the yield traits (Table 3). This suggests that while there is genetic variation among the 22 cultivars tested for yield traits (grain mass, straw yield and harvest index), the variation does not impact their response AWD. It can be concluded that most traits or genes that maximize yield under AWD are the same as those that maximize yield in conventional CF paddy irrigation, which means that current high yielding varieties should perform well (relative to other cultivars) under AWD cultivation as they already do under CF paddy cultivation.

Straw nitrogen concentration was reduced by AWD when compared to CF at two of the three sites. It has been suggested that AWD can affect the fate of nitrogen in paddy fields (Tan et al. 2015). Those authors demonstrated that nitrogen losses due to volatilization and denitrification would increase under AWD compared to CF, using a simulation model of water movement, transport, and transformation. These nitrogen losses are due to the intensified nitrification‐denitrification processes caused by a high concentration of ammonium ions and the cycling between anaerobic and aerobic condition in the soil (Tan et al. 2015). In our study, it could be proposed that more nitrogen was lost in the AWD treatment due to volatilization and denitrification, which directly caused a decrease in plant nitrogen accumulation.

In both the grain and straws of the rice plants grown under AWD at the Madhupur and Mymensingh, the concentration of phosphorus was lower than in the plants grown under CF. This would be expected under anaerobic conditions, because redox‐sensitive mineral constituents (e.g., iron and manganese) release associated (adsorbed or co‐precipitated) phosphorus anions making P more available in the CF treatment. In addition, anaerobic conditions could also result in a release of P from the organic fraction. The difference between soil chemistry under the CF (submerged anaerobic soil) and AWD (soil which is fluctuating between submerged and aerobic soil), could explain a number of the observed effects of AWD on straw and grain element concentrations. The concentration of iron, arsenic, and manganese would be expected to decrease in the soil solution under aerobic conditions, whereas the concentrations of cadmium and copper would be expected to increase under oxidizing conditions (Rinklebe et al. 2016).

For some of the elements at some of the sites, these soil effects can be seen in the plants but this is not the case for all elements at all sites (Tables 4, 5; Figs. 4, 5). While all sites underwent AWD treatment, it appears that the effect of AWD compared to CF was almost negligible at the Rajshahi site for all traits measured. There was no significant difference between the AWD and CF treatments for any of the straw and grain elements measured. In addition, the only yield trait significantly affected by AWD treatment in Rajshahi was straw biomass (Fig. 1a). Because the same AWD treatment was applied across all sites the major difference between sites was, therefore, the soil properties and environmental conditions at each field site. In addition to noting the soil properties of the Rajshahi site, it may be significant that the plants grew much more in this site (higher grain and straw). Both of these factors should be investigated further.

As flowering time was only measured in a single replicate at Mymensingh and not at the other sites, it is not possible to determine the impact that AWD has on flowering time. As there was a range in flowering time for the cultivars and flowering occurred prior to the last AWD cycle finishing, the data can be used to explore if this had an impact on the grain concentration of elements. During the later parts of the AWD cycle, the soil chemistry should change as it moves from an anaerobic soil to an aerobic soil, and this will affect the availability of elements to the plants (Rinklebe et al. 2016). For example, under anaerobic conditions, arsenic availability will be greater than under aerobic conditions, and it has been demonstrated that this impacts the accumulation of arsenic within rice grains, with plants grown under anaerobic conditions have an order of magnitude higher grain arsenic (Xu et al. 2008; Norton et al. 2012a,b, 2013). It is also proposed that the arsenic grain is filled directly from pools of arsenic accumulated by the plant rather than remobilization of arsenic from leaves (Carey et al. 2011). Therefore, it could be hypothesized that plants flowering earlier (when the AWD cycle had not finished) would be exposed to periods of dry (aerobic soil) with lower concentrations of mobile arsenic and therefore would accumulate lower concentrations of arsenic in their grains. However, this was not the case and in fact for arsenic it was the opposite (Figure S3). It is already known that there are strong cultivar differences in elemental concentration in grains (e.g., Jiang et al. 2008; Norton et al. 2010a), it makes the unraveling of the observation of the relationships between flowering time and grain element concentration difficult. Further work on the timing of AWD and how this effects elements is essential, as not only was arsenic effected, but the key nutritional element zinc. However, grain zinc concentration was correlated to flowering time in both AWD and CF grown plants indicating that this is directly related to flowering time rather than the impact that AWD has.

Despite the lack of response to AWD treatment at Rajshahi, the AWD treatment affected the overall concentration of a large number of elements within both the straw and the grains of the rice plants. There were also cultivar differences for a majority of the elements measured in straw and all the elements except for chromium measured in the grains. However, only grain cadmium concentration was affected by the interaction between treatment × cultivar and site × treatment × cultivar. This suggests that for grain cadmium, the genetic mechanism responsible for grain concentration is different when plants are grown under AWD and CF. This means that the genes and quantitative trait loci that have been shown to regulate cadmium accumulation under paddy conditions (Isikawa et al. 2010; Norton et al. 2010b; Zhang et al. 2014; Huang et al. 2015) may not be applicable under AWD. The accumulation of cadmium in grains has been highlighted as a potential risk to human health in rice crops (Meharg et al. 2013). Understanding the mechanisms that regulate the accumulation of cadmium in rice will be beneficial, especially an understanding of the impact that AWD has on the uptake and accumulation mechanisms. For all the other grain elements, the results suggest that progress in regulating the accumulation of these elements (to reduce toxic arsenic or increase beneficial Fe and Zn, for example) would still be equally applicable under AWD cultivation.

In conclusion, AWD in our study has been shown to affect some components of yield and element concentration in plant tissues when rice was grown at three different field sites, but the response differed between sites as indicated by site × treatment interactions. These interactions did not reflect conflicting direction of responses for different sites, rather differences in the magnitude of response in that some sites (especially Rajshahi) did not respond to AWD for some measured traits. The impact that AWD had on the concentration of elements in the grain indicates that AWD may be useful for reducing grain arsenic but care should be taken for areas with high soil available cadmium. Importantly, only grain cadmium concentration was significantly affected by the interaction between treatment and cultivar. This suggests that breeding rice for CF conditions will be equally effective for producing cultivars suited to AWD. Probably it is only if cadmium accumulation is potentially problematic for a specific region that breeding efforts will need to be tailored to AWD.

## Conflict of Interest

None declared.

## Supporting information


**Figure S1.** Straw element traits for the 22 cultivars grown at the three different sites under AWD and CF.

**Figure S2.** Grain element traits for the 22 cultivars grown at the three different sites under AWD and CF.

**Figure S3.** Relationship between flowering time and grain element concentration at the Mymensingh field site.

**Table S1.** Soil properties at the three field sites.

**Table S2.** Mean data for each of the cultivars grown under the different water treatments at each site.
