# Supplementary figures and images for: Biomass and elemental concentrations of 22 rice cultivars grown under alternate wetting and drying conditions at three field sites in Bangladesh
Source: Food Energy Secur. 2017 Jun 15;6(3):98–112. doi: 10.1002/fes3.110 (PMC5599981; doi:10.1002/fes3.110)

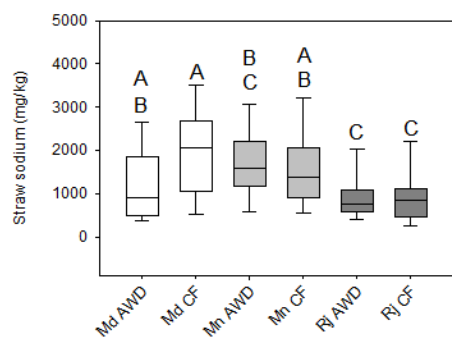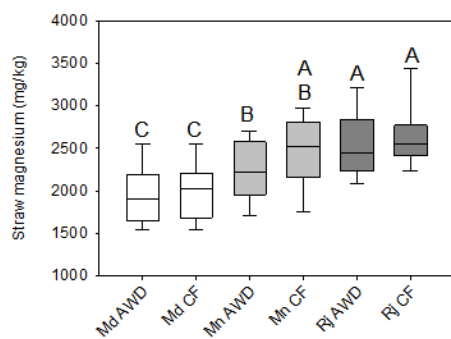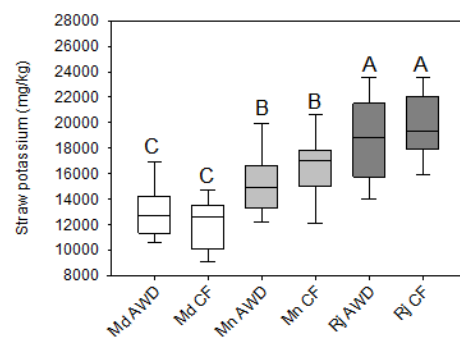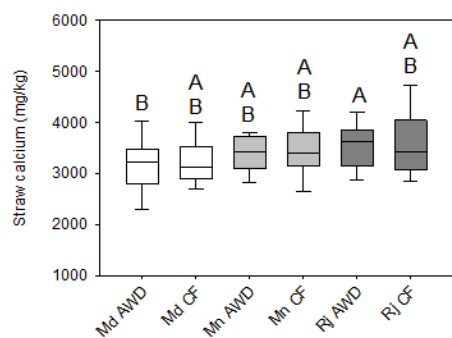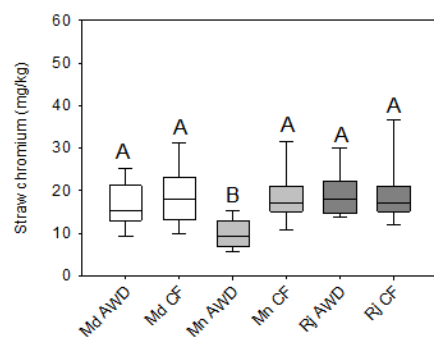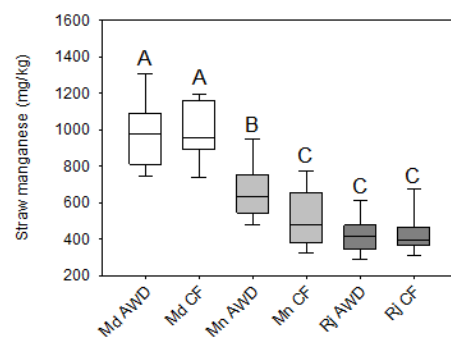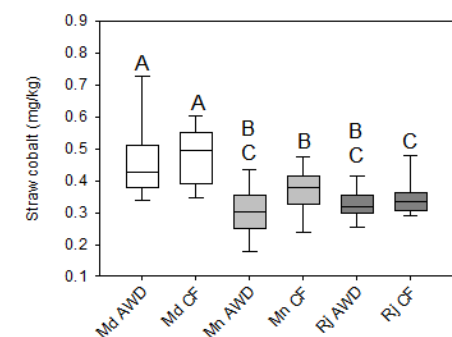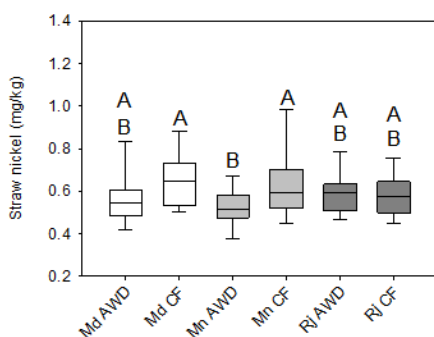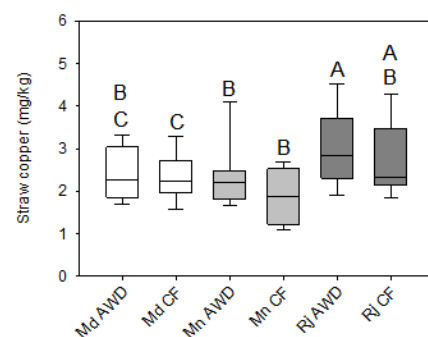

Supplement: Supplementary file 1 — Figure S1. Straw element traits for the 22 cultivars grown at the three different sites under AWD and CF. [file FES3-6-98-s001.pdf]

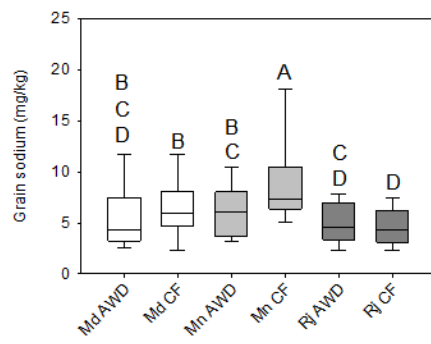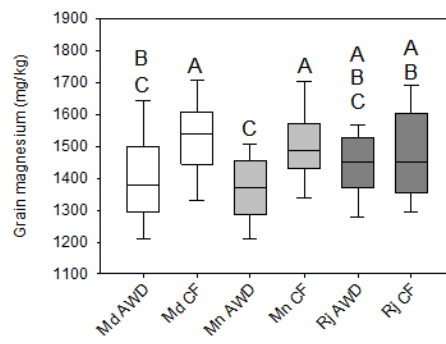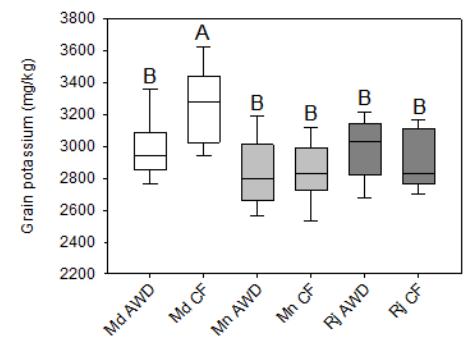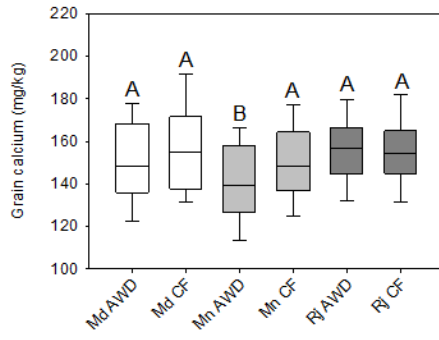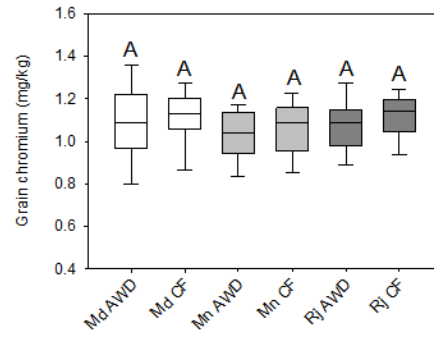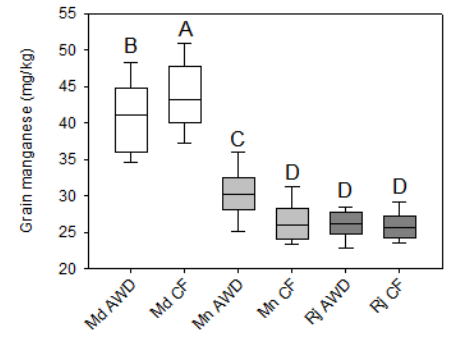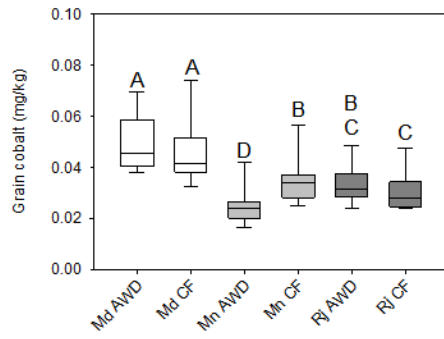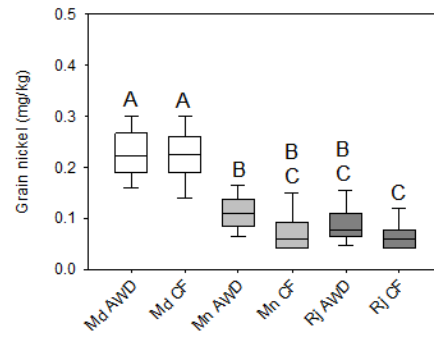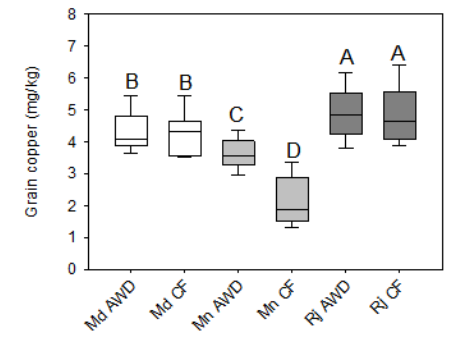

Supplement: Supplementary file 2 — Figure S2. Grain element traits for the 22 cultivars grown at the three different sites under AWD and CF. [file FES3-6-98-s002.pdf]

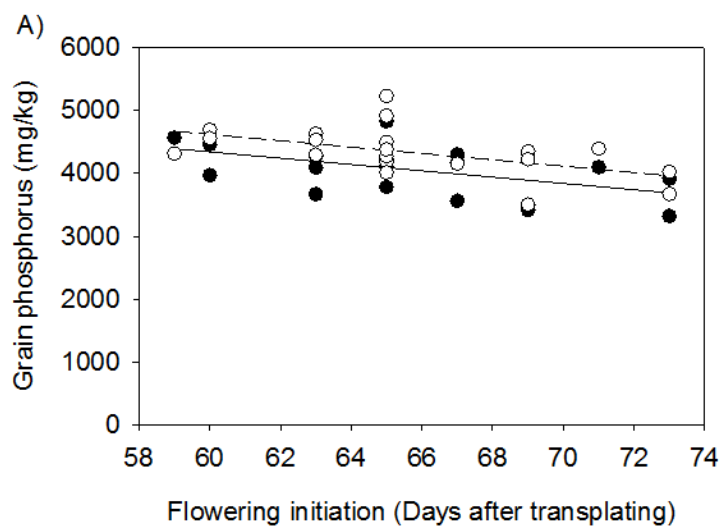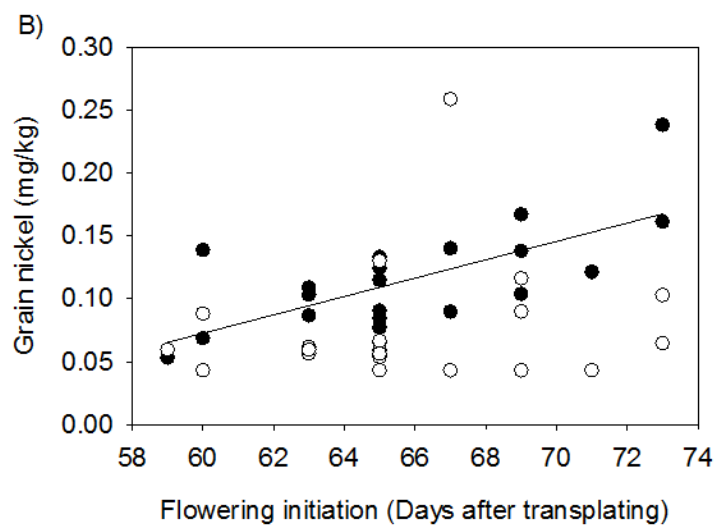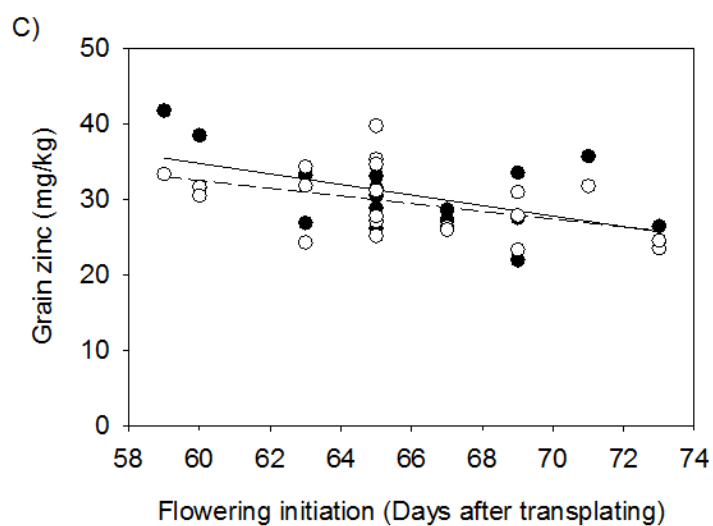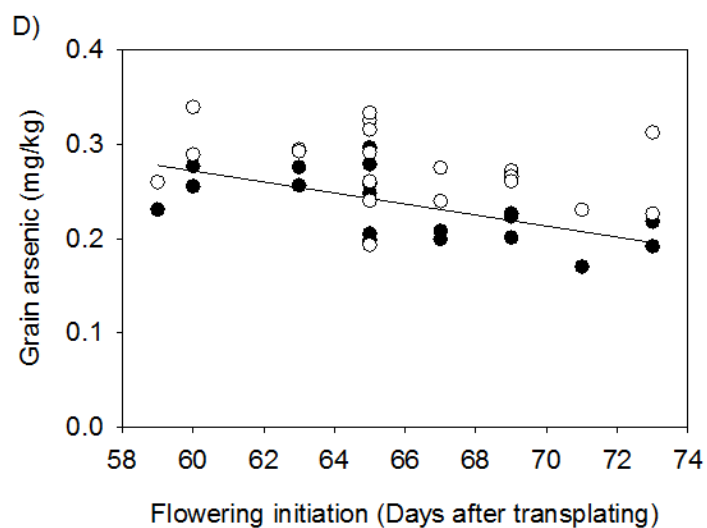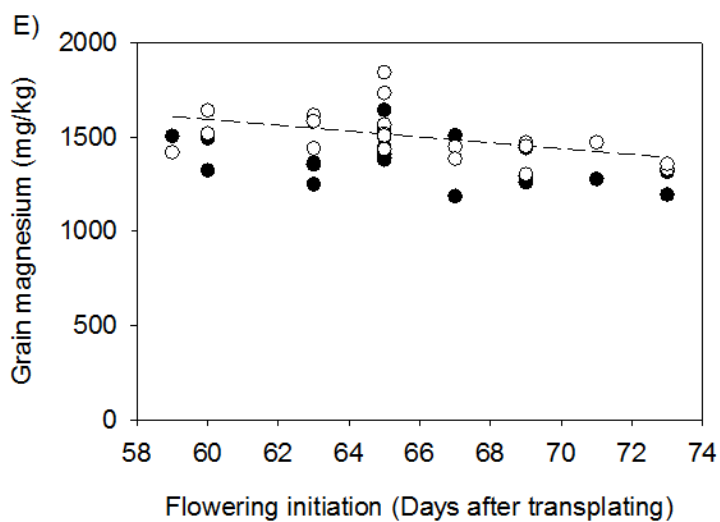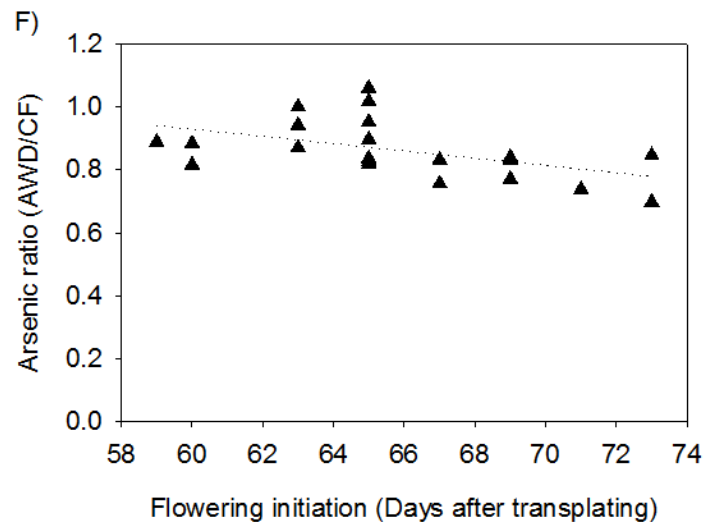

Supplement: Supplementary file 3 — Figure S3. Relationship between flowering time and grain element concentration at the Mymensingh field site. [file FES3-6-98-s003.pdf]
