# Supplementary material for: Biomass and elemental concentrations of 22 rice cultivars grown under alternate wetting and drying conditions at three field sites in Bangladesh
Source: Food Energy Secur. 2017 Jun 15;6(3):98–112. doi: 10.1002/fes3.110 (PMC5599981; doi:10.1002/fes3.110)
Supplement: Supplementary file 4 — Table S1. Soil properties at the three field sites. [file FES3-6-98-s004.docx]

Supplementary table 1. Soil properties at the three field sites.

|  | Mymensingh | Madhupur | Rajshahi |
| --- | --- | --- | --- |
| Soil texture class | Silty clay loam | Clay | Clay loam |
| Soil pH | 6.7 | 6.0 | 7.7 |
| Organic Carbon (%) | 1.7 | 1.2 | 1.4 |
| Nitrogen (%) | 0.16 | 0.11 | 0.12 |
